# MiR-22-3p Inhibits Proliferation and Promotes Differentiation of Skeletal Muscle Cells by Targeting IGFBP3 in Hu Sheep

**DOI:** 10.3390/ani12010114

**Published:** 2022-01-04

**Authors:** Shan Wang, Xiukai Cao, Ling Ge, Yifei Gu, Xiaoyang Lv, Tesfaye Getachew, Joram M. Mwacharo, Aynalem Haile, Wei Sun

**Affiliations:** 1 College of Animal Science and Technology, Yangzhou University, Yangzhou 225009, China; sunnyshan5233@163.com (S.W.); gl1024winnie@163.com (L.G.); feilin133082@163.com (Y.G.); 2 Joint International Research Laboratory of Agriculture, Agri-Product Safety of Ministry of Education of China, Yangzhou University, Yangzhou 225009, China; cxkai0909@163.com (X.C.); dx120170085@yzu.edu.cn (X.L.); 3 International Centre for Agricultural Research in the Dry Areas, Addis Ababa 999047, Ethiopia; t.getachew@cgiar.org (T.G.); j.mwacharo@cgiar.org (J.M.M.); a.haile@cgiar.org (A.H.)

**Keywords:** Hu sheep, skeletal muscle cells, proliferation, differentiation, miR-22-3p, *IGFBP3*

## Abstract

**Simple Summary:**

The meat production of Hu sheep affects the mutton supply, and how to increase the meat production is the primary focus of genetic breeding. Therefore, in order to explore the key factors affecting the proliferation and differentiation of Hu sheep skeletal muscle cells, we performed functional verification of miRNA at the cellular level. Our findings are helpful to clarify the molecular regulation mechanism of proliferation and differentiation of skeletal muscle cells, which will benefit the molecular breeding of Hu sheep.

**Abstract:**

The growth and development of skeletal muscle require a series of regulatory factors. MiRNA is a non-coding RNA with a length of about 22 nt, which can inhibit the expression of mRNA and plays an important role in the growth and development of muscle cells. The role of miR-22-3p in C2C12 cells and porcine skeletal muscle has been reported, but it has not been verified in Hu sheep skeletal muscle. Through qPCR, CCK-8, EdU and cell cycle studies, we found that overexpression of miR-22-3p inhibited proliferation of skeletal muscle cells (*p* < 0.01). The results of qPCR and immunofluorescence showed that overexpression of miR-22-3p promoted differentiation of skeletal muscle cells (*p* < 0.01), while the results of inhibiting the expression of miR-22-3p were the opposite. These results suggested that miR-22-3p functions in growth and development of sheep skeletal muscle cells. Bioinformatic analysis with mirDIP, miRTargets, and RNAhybrid software suggested *IGFBP3* was the target of miR-22-3p, which was confirmed by dual-luciferase reporter system assay. *IGFBP3* is highly expressed in sheep skeletal muscle cells. Overexpression of *IGFBP3* was found to promote proliferation of skeletal muscle cells indicated by qPCR, CCK-8, EdU, and cell cycle studies (*p* < 0.01). The results of qPCR and immunofluorescence experiments proved that overexpression of *IGFBP3* inhibited differentiation of skeletal muscle cells (*p* < 0.01), while the results of interfering *IGFBP3* with siRNA were the opposite. These results indicate that miR-22-3p is involved in proliferation and differentiation of skeletal muscle cells by targeting *IGFBP3*.

## 1. Introduction

Hu sheep is a Chinese local breed, famous for its high reproductivity worldwide. Today, improving meat production of Hu sheep is necessary for the sheep industry in China. Probing the molecular mechanism underlying the proliferation and differentiation of skeletal muscle cells can provide useful clue for this problem.

MiRNA is a non-coding RNA with a length of about 22 nt. The seed region of miRNA can inhibit transcription and translation by targeting and binding mRNA, hence affecting cell proliferation, apoptosis at different developmental stages [1]. To date, several important miRNAs have been identified for regulating muscle growth and development, such as miR-205, miR-126, miR-60, miR-75, miR-133, miR-499, etc. [2]. *MyoD1*, a marker gene of muscle differentiation, is regulated by miR-1 and miR-206 [3,4]. MiR-192 targets myogenic regulator *RB1*, inhibiting the proliferation and promoting the differentiation of Hu sheep skeletal muscle cells [5]. MiR-128 participates in the regulation of the CDS region of myostatin and inhibits the proliferation and promotes differentiation of C2C12 cells [6]. MiR-143, miR-696, miR-34b, etc., are involved in the growth process of skeletal muscle cells, including proliferation and differentiation [7,8,9]. However, there are still a lot of miRNAs without functional verification in terms of muscle growth and development in sheep.

MiR-22-3p has been found as one of the differentially expressed miRNAs during Hu sheep muscle development [10]. A recent study showed that miR-22-3p treats fibrous cataract by targeting *HDAC6* [11]. Another report indicated miR-22-3p inhibits proliferation and promotes differentiation of porcine skeletal muscle cells [12]. Studies related to C2C12 cells showed that miR-22-3p inhibits proliferation and promotes differentiation of C2C12 cells, meanwhile promoting the transition from fast-twitch to slow-twitch [13,14]. Although quite a few research works have studied on miR-22-3p, there are no reports on skeletal muscle cells in Hu sheep.

The IGFs are related to muscle growth and development. As IGF binding proteins, the IGFBPs also play an important role in biology process. *IGFBP2* induces the proliferation and invasion of glioma cells through the β1/ERK signaling pathway, indicating that *IGFBP2* can be used as a potential therapeutic target for gliomas [15]. *IGFBP1* and *IGFBP2* are regulated by insulin, which affect glucose tolerance, participate in glucose metabolism and lipid metabolism, and have a therapeutic effect on obesity [16]. Studies have shown that *IGFBP3* can be used as a therapeutic target for lung adenocarcinoma metastasis to the brain [17]. Increasing expression of *IGFBP3* could promote the formation of endothelial bone in rats fed with eleutherococcus extract mixture (EEM) [18]. The enhanced transcription of *IGFBP3* accumulates the abundance of *IGF1*, which affects the growth and metabolism of mice [19]. Under repeated acute stress, the expression levels of *IGFBP3* and *IGF1* in pig blood were elevated, and the IGF system was activated at this time, indicating that *IGFBP3* is involved in acute physiological stress response, inflammation pathways, and energy metabolism pathways [20]. Although there are many studies on *IGFBP3* in cancer and metabolic pathways, its roles in the growth and development of skeletal muscle in Hu sheep are not yet known. In this study, we speculated that miR-22-3p could have a regulatory effect on the growth and development of skeletal muscle cells in Hu sheep.

In summary, to explore the function of miR-22-3p regulating skeletal muscle cells of Hu sheep, we carried out qPCR, CCK-8, EdU, cell cycle, and immunofluorescence studies and found overexpression of miR-22-3p inhibited differentiation and promoted proliferation of skeletal muscle cells. We identified *IGFBP3* as one of its target gene by Dual-luciferase reporter system assay. Our results suggested that miR-22-3p plays a significant role in the growth process of skeletal muscle cells by targeting *IGFBP3* in Hu sheep, making miR-22-3p as a molecular marker for breeding.

## 2. Material and Methods

### 2.1. Ethics Statement

All experimental procedures were strictly in accordance with the management measures of experimental animals in Jiangsu Province (License Number: 45). All animal procedures used in this study were approved by the Ethics Committee for Animal Experiments of Yangzhou University (No. 202103279) and were performed in accordance with the Guidelines for Animal Experimentation of Yangzhou University (Yangzhou, China).

### 2.2. Cell Culture

Primary skeletal muscle cells were isolated from three 56-day fetal sheep according to the previous method [21] and cultured in Dulbecco’s Modified Eagle Medium (DMEM, Gibco, Grand Island, NE, USA) supplemented with 20% fetal bovine serum (FBS, Gibco, Grand Island, NE, USA) and 1% Penicillin streptomycin mixture 100× (Solarbio, Beijing, China). HEK293T cells were obtained from American Type Culture Collection (ATCC, Manassas, VA, USA) and cultured in DMEM supplemented with 10% FBS and 1% Penicillin streptomycin mixture 100×. Cells were cultured in 37 °C with 5% CO_2_.

### 2.3. Plasmid Construction and Cell Transfection

The coding region of *IGFBP3* and pcDNA3.1 (+) vector were double digested with restriction enzymes *Xho* I and *EcoR* I (Takara, Kusatsu, Shiga, Japan), and the 3′UTR of *IGFBP3* and psi-check2 plasmid (Youbio, Changsha, China) were double digested with restriction enzymes *Xho* I and *Not* I (Takara, Kusatsu, Shiga, Japan). Enzyme products were recovered by MiniBEST Agarose Gel DNA (Takara, Kusatsu, Shiga, Japan), connected by solution I ligase (Takara, Kusatsu, Shiga, Japan) and transformed using Trelief™ 5α Chemically Competent Cell (Tsingke, Nanjing, China). The recombinant vectors were extracted using an Endofree Mini plasmid kit II (Tiangen, Beijing, China) Extraction kit, followed by confirmation with enzyme digestion sequencing. The small interference sequences of *IGFBP3* were designed and synthesized by Genepharma Co., Ltd. (Suzhou, China).

When cells grew to 60% confluence, we transfected inhibitor and mimics of miR-22-3p, siRNA, or recombinant vector of *IGFBP3* to perform interference or overexpression assays with jetPRIME transfection reagent (Polyplus, New York, NY, USA), with at least three replicates, respectively. After 24 h of transfection, the corresponding experiment was carried out. Specific sequences were shown in Table 1.

### 2.4. RNA Preparation and qPCR

When cells grew to 80% confluence, cellular RNA was extracted with reference to TRIzol total RNA extraction reagent (Beyotime, Shanghai, China). Reverse transcription was followed by a one-step reverse transcription kit (Tiangen, Beijing, China). Products were used to conduct qPCR using a CFX96 fluorescence quantitative instrument according to the 2× TSINGKE^®^ Master qPCR mix (Tsingke, Nanjing, China) reagent manual. Primers were synthesized by Sangon biotech Co., Ltd. (Shanghai, China). Specific sequences were shown in Table 2.

### 2.5. CCK-8 Cell Counting Kit Assay

Skeletal muscle cells were cultured in 96-well plates until 60% cell confluence. Transfection was performed with 24-h incubation. Each group had 6 parallel wells, and CCK-8 Cell viability was tested at 0 h, 24 h, 48 h, and 72 h. Specific steps of CCK-8 reagent (Beyotime, Shanghai, China,) were as follows: to each well was added 10 μL of CCK-8 reagent, and cells were incubated in 37 °C for 2 h. Finally, cells were measured by a microplate reader (OD value at 450 nm).

### 2.6. Cell Cycle Kit Assay

When cells grew to 80% confluence, they were re-seeded onto 6-well plates (1 mL/well). Transfection was performed under 60% cell confluence, followed by 24h incubation. Cells were digested with 0.25% trypsin and centrifuged at 1000× *g* for 5 min with 1 mL PBS. Each well was added 1 mL pre-cooled 70% ethanol, mixed by pipetting, and was fixed at 4 °C for 24 h. Then, cells were rinsed by pre-cooled PBS and centrifuged. Next, a solution of propidium iodide was prepared according to the Beyotime Cell Cycle Kit (Beyotime, Shanghai, China). To each tube of cells was added 0.5 mL staining solution, incubated at 37 °C for 30 min in the dark, and detected by flow cytometric.

### 2.7. 5-Ethynyl-2′-Deoxyuridine (EdU) Assay

When cells grew to 80%, they were digested with 0.25% trypsin, added appropriate complete medium to prepare cell suspension, then distributed cells in 96-well plates (100 μL/well), and placed in 5% CO_2_, 37 °C constant temperature incubator. When they grew to 60%, we performed transfection. Each group had 6 parallel wells, and the EdU test was performed when the cells grew to 80%. Specific methods refer to the instruction of EdU cell proliferation detection (Ribobio, Guangzhou, China) and observation with a fluorescent inverted microscope.

### 2.8. Immunofluorescence Staining

When cells grew to 80%, they were distributed to 24-well plates (500 μL/well). When they grew to 60%, we performed transfection. At the same time, cells were cultured in DMEM supplemented with 2% horse serum (Solarbio, Beijing, China) and 1% Penicillin streptomycin mixture 100 ×. There were 3 parallel wells in each group, and the cellular immunofluorescence test was carried out after 4–5 days of induced differentiation. Specific steps of immunofluorescence staining were as follows: The cells were washed twice with PBS, 3–5 min each time, and to each well was added 300 μL 4% paraformaldehyde (Solarbio, Beijing, China); they were fixed for 30 min. Then, they were washed in the same way and permeated with 0.5% Triton X-100 (Solarbio, Beijing, China) for 20 min, and added 300 μL of 1% BSA (Solarbio, Beijing, China) to block for 1 h. Next, each well added 200 μL primary antibody MyH3 (1:400) (Affinity, Changzhou China) at 4 °C overnight, then added 200 μL secondary antibody (1:1000) (Abconal, Wuhan, China), incubating for 2 h. Finally, each well added 200 μL DAPI (5 μg/mL) (Beyotime, Shanghai, China) for staining in 5 min. Fluorescence inverted microscope was used to observe cell differentiation and myotube formation.

### 2.9. Dual-Luciferase Reporter System Assay

When HEK293T cells grew to 80%, they were distributed to 24-well plates (500 μL/well). When they grew to 80%, we performed transfection. Each group was in 3 parallel holes. Groups were designed as follows: miR-22-3p mimics-NC + IGFBP3-wild, miR-22-3p mimics + IGFBP3-wild, miR-22-3p mimics -NC+ IGFBP3-mutant, miR-22-3p mimics + IGFBP3-mutant. After 48 h of transfection, the cells were tested according to the Dual Luciferase Reporter Assay Kit (Vazyme, Najing, China). Specific steps were as follows: To each well was added 100 μL 1 × Cell Lysis Buffer, pipetted into a 1.5 mL centrifuge tube, centrifuged at 12,000× *g* for 2 min. Next, to each well was added 100 μL of Luciferase Substrate to the microplate, absorbing 20 μL of the above cell lysate, immediately detecting the activity of the Firefly luciferase reporter gene. To the above reaction solution was added 100 μL Renilla substrate, immediately detecting the activity of the Renilla luciferase reporter gene. The ratio of Renilla fluorescence value to firefly fluorescence value is the relative luciferase activity, and the ratio is statistically analyzed with the ratio of the control well.

### 2.10. Statistical Analysis

Results were presented as the Mean ± SEM. One-way analysis of variance was used to perform variance analysis and significance test. Results were considered significant at *p* < 0.05 and highly significant at *p* < 0.01.

## 3. Results

### 3.1. miR-22-3p Regulates the Proliferation of Skeletal Muscle Cells in Hu Sheep

**Figure 1:**
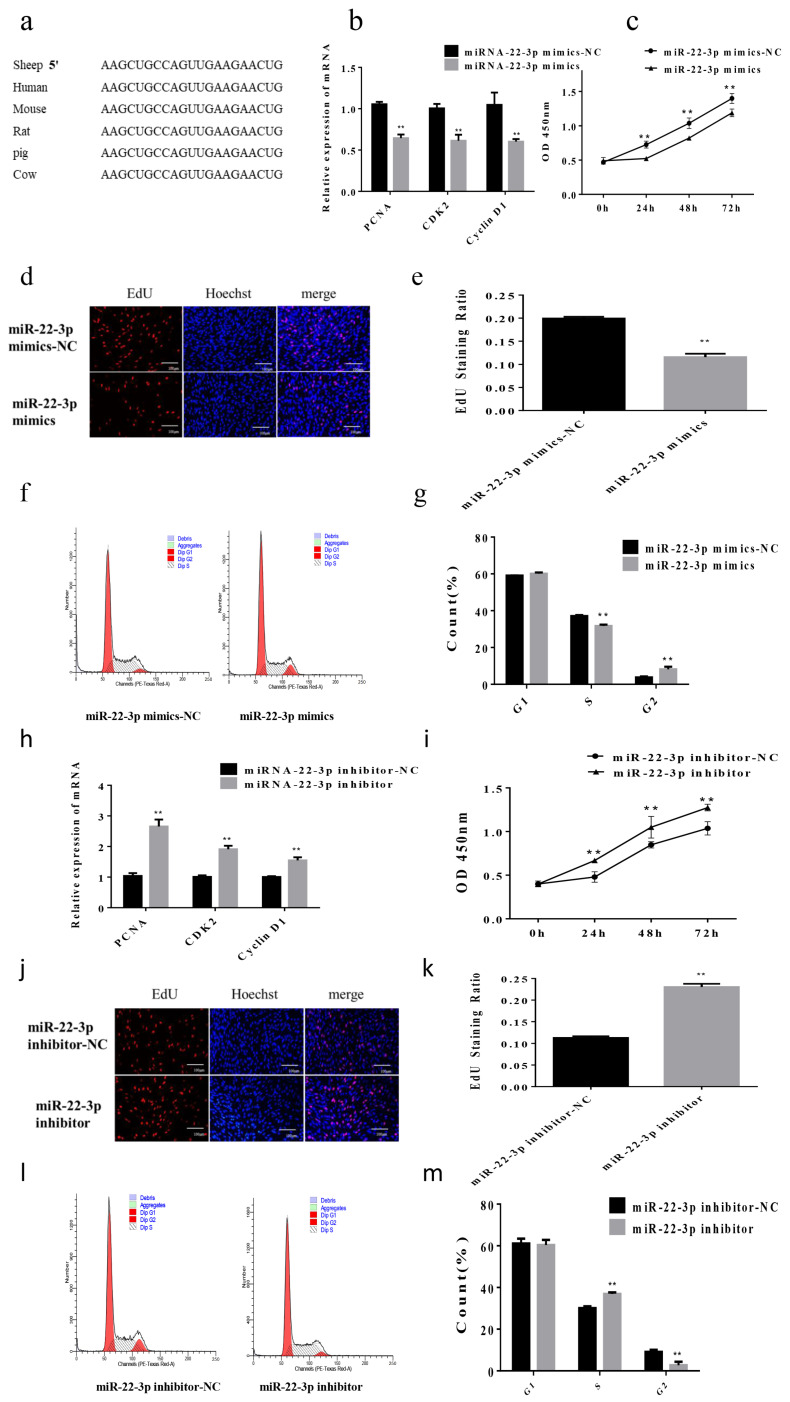
The effect of miR-22-3p on skeletal muscle proliferation of Hu Sheep. (**a**) Homology analysis of mature sequences of miR-22-3p in different species; (**b**) the relative expression of *PCNA, CDK2* and *cyclin D1* after overexpression of miR-22-3p; (**c**) the OD_450_ of all living cells after transfection of miR-22-3p mimics; (**d**,**e**) EdU cell proliferation assay (400×); proliferation rate of transfected miR-22-3p mimics; (**f**,**g**) cell cycle; ratio of skeletal muscle cells at different stages after overexpression of miR-22-3p; (**h**) the relative expression of *PCNA, CDK2* and *cyclin D1* after interference with miR-22-3p; (**i**) the OD_450_ of all living cells after transfection of miR-22-3p inhibitor; (**j**,**k**) EdU cell proliferation assay (400×); cell proliferation rate after transfection of miR-22-3p inhibitor; (**l**,**m**) cell cycle; ratio of skeletal muscle cells at different stages after interference with miR-22-3p. (** compared with the control group, *p* < 0.01).

We used miRBase software to get the sequences of miR-22-3p across diverse species and found that it was highly conserved (Figure 1a). In order to study the effects of miR-22-3p in Hu sheep skeletal muscle cells, we transfected miR-22-3p mimics into the sheep skeletal muscle cells. The qPCR results showed that after overexpression of miR-22-3p, the mRNA expression of proliferation marker genes *PCNA*, *CDK2*, and *cyclin D1* were significantly lower than that of the control group (*p* < 0.01) (Figure 1b). The proliferation status of sheep skeletal muscle cells was detected by CCK-8 reagent, and the OD_450_ value of the miR-22-3p group was significantly reduced (*p* < 0.01) (Figure 1c). The results of EdU staining showed that the number of EdU positive cells was significantly reduced compared with the control group (*p* < 0.01) (Figure 1d,e). In addition, the results of flow cytometry showed that the number of cells in S phase was significantly lower than that of the control group (*p* < 0.01) (Figure 1f,g). Next, we transfected the miR-22-3p inhibitor into the cells and found that the expression levels of *CDK2*, *cyclinD1*, and *PCNA* increased compared with the control group (*p* < 0.01) (Figure 1h). The results of CCK-8 showed that the OD_450_ of the cells increased (*p* < 0.01) (Figure 1i). In addition, the number of EdU positive cells increased significantly (*p* < 0.01) (Figure 1j,k). At the same time, the results of flow cytometry showed that the number of cells in S phase was significantly higher than that of the control group (*p* < 0.01) (Figure 1l,m). The above results indicated that miR-22-3p could inhibit the proliferation of skeletal muscle cells in Hu sheep.

### 3.2. miR-22-3p Regulates the Differentiation of Skeletal Muscle Cells in Hu Sheep

**Figure 2:**
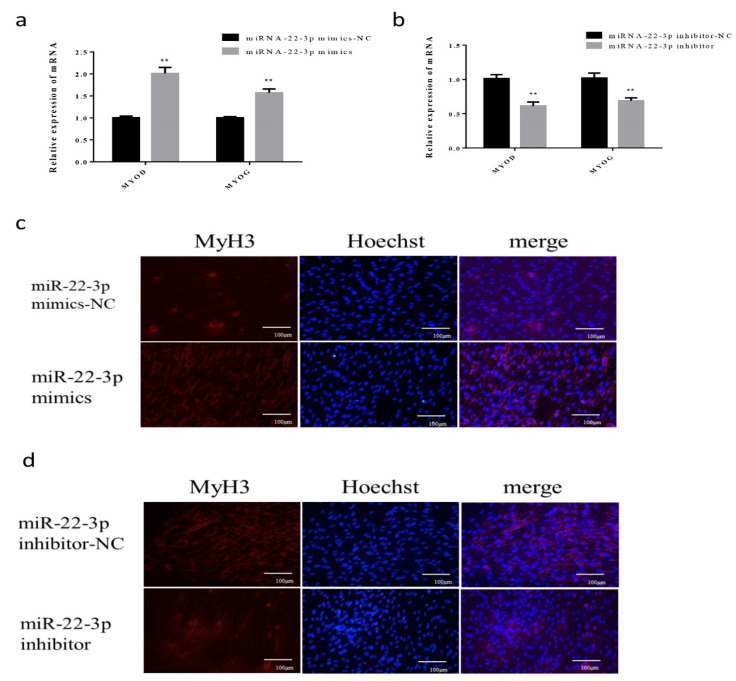
The effect of miR-22-3p on skeletal muscle differentiation of Hu Sheep (**a**) The relative expression of *MyoD* and *MyoG* after overexpression of miR-22-3p; (**b**) the relative expression of *MyoD* and *MyoG* after interference with miR-22-3p inhibitor; (**c**) after overexpression of miR-22-3p, the red fluorescence of *MyH3* was detected; (**d**) the red fluorescence of *MyH3* was detected after interfering with miR-22-3p inhibitor. (** compared with the control group, *p* < 0.01).

After transfection of miR-22-3p mimics and NC, the differentiation marker genes *MyoD* and *MyoG* were significantly increased as indicated by qPCR (*p* < 0.01) (Figure 2a). At the same time, the immunofluorescence results showed that the number of *MyH3* positive myotubes was more than that of the control group (Figure 2c). Next, we transfected the miR-22-3p inhibitor and NC into cells, and the differentiation marker genes, *MyoD* and *MyoG*, were significantly reduced (*p* < 0.01) (Figure 2b). Immunofluorescence results showed that the number of *MyH3* positive myotubes was less than that of the control group (Figure 2d). These results indicated that miR-22-3p could promote the differentiation of skeletal muscle cells in Hu sheep.

### 3.3. miR-22-3p Regu’Lates the Expression of IGFBP3

**Figure 3:**
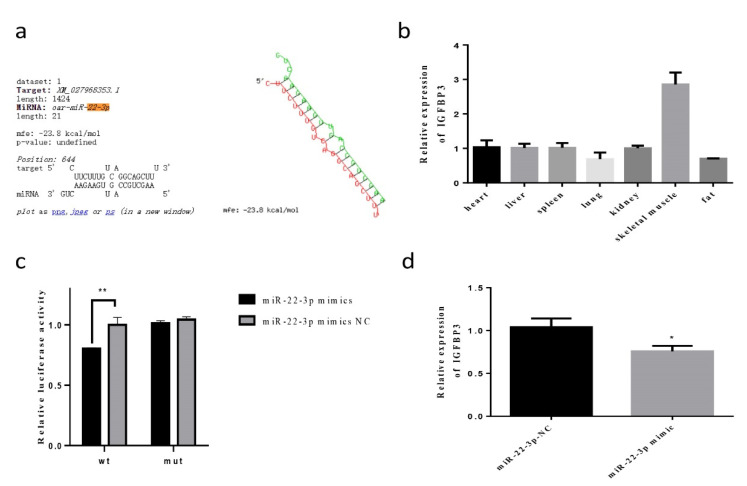
The detection of targeting relationship between miR-22-3p and *IGFBP3* (**a**) RNAhybrid predicted the targeted binding relationship between *IGFBP3* and miR-22-3p; (**b**) the relative expression of *IGFBP3* in tissue expression profile; (**c**); the relative activity of wild-type and mutant miR-22-3p was detected by double luciferase activity; (**d**) the relative expression of *IGFBP3* after transfecting of miR-22-3p mimics. (* compared with the control group, *p* < 0.05, ** compared with the control group, *p* < 0.01).

We used mirDIP and miRTargets software to predict the target genes of miR-22-3p. The result was confirmed by RNAhybrid (Figure 3a). We focused on *IGFBP3* because the function of this gene in sheep skeletal muscle cells is unknown yet, and it is differentially expressed during skeletal muscle development (unpublished RNA-seq data). At the same time, the mirDIP bioinformatic software predicted *IGFBP3* as target with a high score. The tissue expression profile showed that *IGFBP3* had the highest expression in skeletal muscle cells (Figure 3b). The results of the dual luciferase reporter vector showed that the fluorescence activity is reduced (Figure 3c). After transfection of miR-22-3p mimics and NC, the expression of *IGFBP3* was significantly reduced (*p* < 0.05) (Figure 3d).

### 3.4. IGFBP3 Regulates the Proliferation of Skeletal Muscle Cells in Hu Sheep

**Figure 4:**
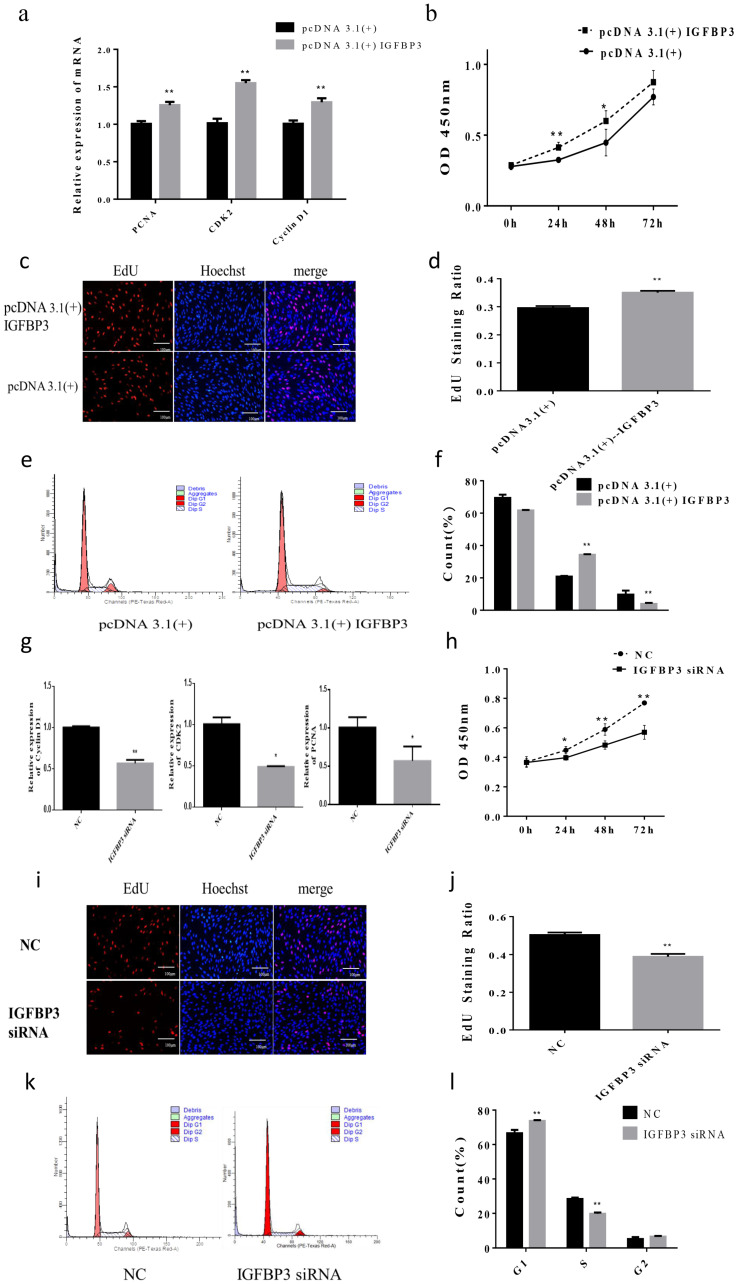
The effect of *IGFBP3* on skeletal muscle proliferation of Hu Sheep. (**a**) The relative expression of *PCNA*, *CDK2*, and *cyclin D1* after overexpression of *IGFBP3*; (**b**) the OD_450_ of all living cells after transfection of *IGFBP3* overexpression vector; (**c**,**d**) EdU cell proliferation assay (400×); proliferation rate of transfected the overexpression of *IGFBP3*; (**e**,**f**) cell cycle; ratio of skeletal muscle cells at different stages after overexpression of *IGFBP3*; (**g**) the relative expression of *PCNA*, *CDK2*, and *cyclin D1* after interference with *IGFBP3*; (**h**) the OD_450_ of all living cells after transfection with interference with *IGFBP3*; (**i**,**j**) EdU cell proliferation assay (400×); cell proliferation rate after transfection of *IGFBP3* inhibitor; (**k**,**l**) cell cycle; ratio of skeletal muscle cells at different stages after interference with *IGFBP3*. (* compared with the control group, *p* < 0.05, ** compared with the control group, *p* < 0.01).

We constructed the overexpression vector of *IGFBP3* and transfected it into skeletal muscle cells. The qPCR results showed that the expression of key genes *CDK2*, *cyclin D1*, and *PCNA* for cell proliferation increased significantly (*p* < 0.01) (Figure 4a). CCK-8 showed that the OD_450_ of the cells was significantly higher than that of the control group after transfection 24 h (*p* < 0.01) (Figure 4b). In addition, the number of EdU positive cells increased significantly (*p* <0.01) (Figure 4c,d). The results of flow cytometry showed that the number of cells in S phase was significantly higher than that of the control group (*p* < 0.01) (Figure 4e,f). Next, we interfered *IGFBP3* mRNA with siRNA. The qPCR results showed that the expression level of *cyclin D1* was extremely significantly reduced (*p* < 0.01), and the mRNA expression of *CDK2* and *PCNA* was significantly reduced (*p* < 0.05) (Figure 4g). CCK-8 showed that the OD_450_ value of the interference group was significantly reduced after 48 h and 72 h compared with the control group (*p* < 0.01) (Figure 4h). EdU staining results showed that the number of positive cells was significantly reduced (*p* < 0.01) (Figure 4i,j). In addition, the results of flow cytometry showed that the number of cells in S phase was significantly lower than that of the control group (*p* <0.01) (Figure 4k,l). These results indicated that *IGFBP3* could regulate proliferation of skeletal muscle cells.

### 3.5. IGFBP3 Regulates the Differentiation of Skeletal Muscle Cells in Hu Sheep

**Figure 5:**
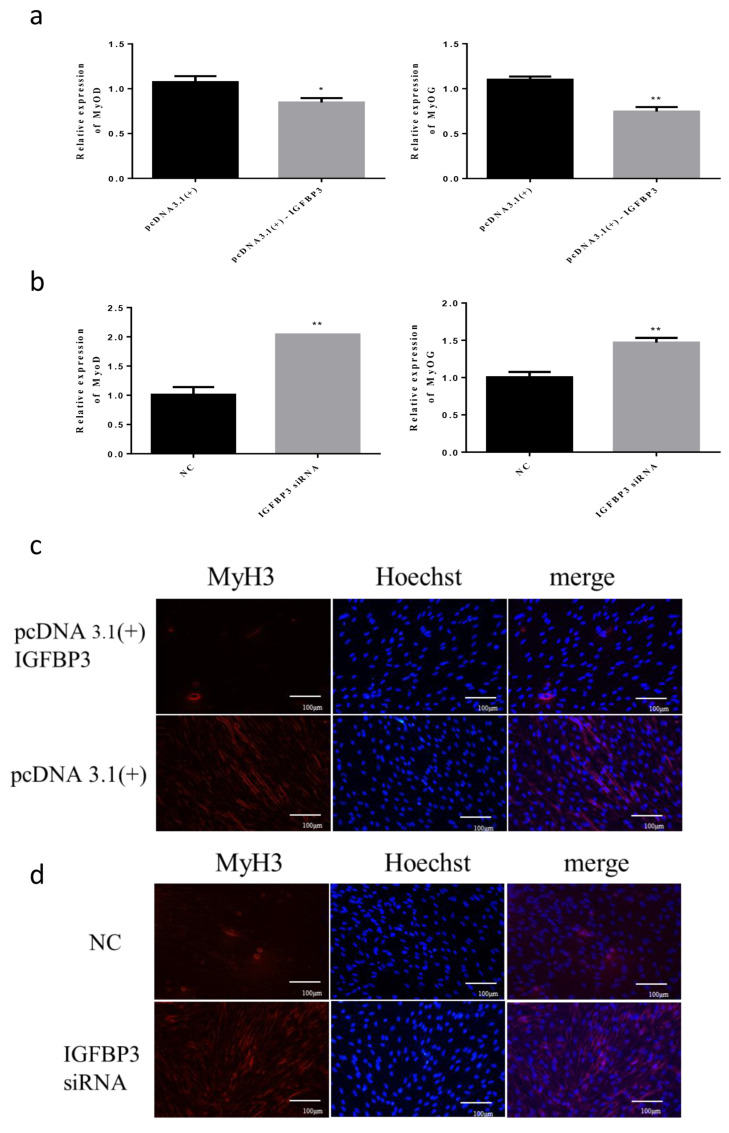
The effect of *IGFBP3* on skeletal muscle differentiation of Hu Sheep. (**a**) The relative expression of *MyoD* and *MyoG* after overexpression of *IGFBP3*; (**b**) the relative expression of *MyoD* and *MyoG* after interference with *IGFBP3*; (**c**) after overexpression of *IGFBP3*, the red fluorescence of *MyH3* was detected; (**d**) the red fluorescence of *MyH3* was detected after interfering with *IGFBP3*. (* compared with the control group, *p* < 0.05, ** compared with the control group, *p* < 0.01).

Transfecting of *IGFBP3* overexpression vector and NC, we found that the differentiation marker gene *MyoD* was significantly reduced (*p* < 0.05), and *MyoG* was extremely significantly reduced compared with the control group (*p* < 0.01) (Figure 5a). At the same time, the immunofluorescence results showed that the number of *MyH3* positive myotubes was less than that of the control group (Figure 5c). Next, we transfected the *IGFBP3* siRNA into skeletal muscle cells, and the differentiation marker genes *MyoD* and *MyoG* were significantly increased (*p* < 0.01) (Figure 5b). Immunofluorescence results showed that the number of *MyH3* positive myotubes was more than that of the control group (Figure 5d). In summary, these results indicated that *IGFBP3* could regulate the proliferation of skeletal muscle cells in Hu sheep.

Through cell proliferation and differentiation verification experiments, we found that overexpression of miR-22-3p could inhibit proliferation and promote differentiation of skeletal muscle cells by targeting *IGFBP3* in Hu sheep. These findings may benefit the understanding of the mechanism of growth and development of skeletal muscle cells at miRNA levels.

## 4. Discussion

MiRNA was first found in nematodes and was named lin-4, and another miRNA named let-7 was found, both of which were about 22 nt in length. These miRNAs can bind to the 3′UTR of mRNA, thereby regulating the development of nematodes [1]. In the following years, miRNAs and multiple potential mechanisms of their binding were discovered in thousands of species, opening up a new world in the field of scientific research. MiRNA is mainly processed and produced in the cytoplasm and transported to the nucleus. It can be used in transcription activation. Studies have revealed that miR-744 functions in the transcription initiation site of *Cyclin B1* under Ago1 participating [22]. There are also related reports that miRNA can target the 5′UTR region of mRNA and inhibit mRNA expression. The classic miRNA regulatory network aims to inhibit mRNA expression by targeting the 3′UTR region of mRNA. In colorectal cancer tissues, tumor transcription factor *Jun* inhibits the transcription of miR-22, and miR-22 targets *TIAM1*. Reducing the expression of miR-22-3p inhibits the proliferation of cancer cells [23,24,25]. In livestock, this classic regulation mechanism has also been reported. Research shows that miR-1 and miR-206 target *PAX7*, i.e., inhibiting the proliferation of skeletal muscle by inhibiting the expression of these two miRNAs [26], thereby regulating muscle growth and development.

Although many miRNAs have been reported in domestic animals, there are still a lot of miRNAs that require further verification in terms of muscle growth. Since the number of myotubes is constant before birth, we focus on miRNAs with low expression before birth and high expression after birth. MiR-22-3p has been detected in the longissimus dorsi muscle of sheep and the expression of miR-22-3p is the lowest at 60 days of pregnancy and the highest at 360 days after birth [10]. Cancer literatures proved that miR-22-3p targets *SP1*, inhibits the expression of downstream genes *CCND1* and *BCL2*, thereby inhibiting the growth of liver cancer cells, and the low expression of miR-22-3p is associated with metastatic liver cancer [27,28]. Hsa-miR-22-3p serves as the target of *DGCR5*, reducing its expression to inhibit the occurrence of lung cancer [29]. It is documented that the transfection of miR-22-3p inhibits the proliferation of skeletal muscle cells and promotes differentiation in porcine skeletal muscle cells [12]. Overexpression of miR-22-3p inhibits the proliferation of C2C12 cells (mouse myoblast) and promotes the differentiation of muscle fibers, promoting the transition of mouse C2C12 myotube fiber type from fast-twitch to slow-twitch [13,14]. However, the function of miR-22-3p regulating skeletal muscle cells in Hu sheep has not been reported yet. What is exciting is that we found that the mature sequence of miR-22-3p is completely conserved among different species, which further supported our assumption. Thence, we carried out experiments in Hu sheep skeletal muscle cells.

In order to explore the function of miR-22-3p in Hu sheep skeletal muscle cells, we adopted qPCR, CCK-8, EdU, cell cycle, and immunofluorescence assays. Through the preliminary verification by qPCR, the proliferation marker genes of the miR-22-3p mimics transfected group were significantly reduced, indicating that overexpression of miR-22-3p inhibited the proliferation of skeletal muscle cells. *PCNA*, *CDK2*, and *cyclin D1*, the well accepted proliferation marker genes, were used in study to detect whether skeletal muscle cells are in a proliferating state [30]. However, it has been reported that *CDK4*, *cyclin E2*, and *E2F1* are used as pygmy killer whale skin fibroblasts proliferation genes [31], which may be due to the genes in specific expression of different species and different tissues. However, we still want to continue to explore the influence of the downstream genes of miR-22-3p on the growth and development of skeletal muscle cells. Through qPCR and immunofluorescence, we found that the differentiation marker genes were significantly up-regulated after overexpression of miR-22-3p. *MyoD* and *MyoG*, differentiation markers, have also been used in the research of fetal bovine skeletal muscle [30]. These results indicated that overexpression of miR-22-3p inhibited the proliferation and promoted differentiation of skeletal muscle cells. However, we still want to explore the influence of the downstream regulatory elements of miR-22-3p regulating the growth and development of skeletal muscle cells in Hu sheep.

Bioinformatic analysis with mirDIP, miRTargets, and RNAhybrid software predicted the targeting relationship between downstream genes and miR-22-3p. Previous study has identified *HDAC6* as a downstream target gene of miR-22-3p in lens epithelium cells [11]. Here, we predicted it through miRTargets software, but its score is not high. What is exciting is that we found *IGFBP3* has a high score in miRTargets, mirDIP, and RNAhybrid software, and the seed sequence of miR-22-3p completely binds to the 3′UTR region of *IGFBP3*, so we focused on *IGFBP3*. A large number of literature reports that IGFs are indispensable in the growth and development of skeletal muscle. Studies have shown that IGFs promote the proliferation and differentiation of muscle cells, as well as regulate each other with *MRF* and *MyoD* to improve muscle hypertrophy and regeneration [32,33]. Studies showed that *IGFBP1* activates ERK1/2 pathway to promote the proliferation of smooth muscle cells (SMCs) by regulating *IGF1* [34]. *IGFBP6* inhibits the expression of *IGF2* and activates the MAPK pathway to promote muscle differentiation, and the activation of this pathway does not require *IGF-1R* or insulin receptor (IR) to participate [35]. These findings reveal that the IGFBPs are involved in the growth and development of muscles. Based on this, we speculated that *IGFBP3* has a similar effect on Hu sheep skeletal muscle. The tissue expression profile preliminarily certificated that *IGFBP3* is highly expressed in skeletal muscle cells. Moreover, a large number of research reports illustrate that *IGFBP3* is currently only studied in diseases. *IGFBP3* activates the *XBP1/IGFBP3/MMP-9* pathways to regulate the invasion and metastasis of non-small cell lung cancer (NSCLC) cells [36]. Studies have shown that Vi4 and miRNA-185-5p competitively combine with *IGFBP3* to promote neuronal cell proliferation and reduce the risk of neonatal hypoxic ischemic encephalopathy (HIE) [37]. In view of the fact that no research related to muscles has been carried out, we made a bold attempt to deeply explore the effect of *IGFBP3* in skeletal muscle cells of Hu sheep. Experiments were carried out by qPCR, CCK-8, EdU, cell cycle, and immunofluorescence studies, and these studies indicated that overexpression of *IGFBP3* promoted proliferation and inhibited differentiation of skeletal muscle cells in Hu sheep.

## 5. Conclusions

In summary, our results indicated that overexpression of miR-22-3p inhibited proliferation and promoted differentiation of skeletal muscle cells by targeting *IGFBP3* in Hu sheep. The expression activity of miR-22-3p is low, which indicates that the Hu sheep muscle cell proliferation efficiency is high, and miR-22-3p is used to identify the inflection point of Hu sheep muscle growth. Our findings are helpful to clarify the molecular regulation mechanism of proliferation and differentiation of skeletal muscle cells, which will benefit the molecular breeding and theoretical basis to mutton producers of Hu sheep.

## Figures and Tables

**Table 1:** The information of synthetic sequence.

Gene/miRNA Name	Forward Primer (5′-3′)	Reverse Primer (5′-3′)
*IGFBP3* NC	UUCUCCGAACGUGUCACGUTT	ACGUGACACGUUCGGAGAATT
*IGFBP3* siRNA	GCACAGACACCCAGAACUUTT	AAGUUCUGGGUGUCUGUGCTT
miR-22-3p mimics NC	UUCUCCGAACGUGUCACGUTT	ACGUGACACGUUCGGAGAATT
miR-22-3p mimics	AAGCUGCCAGUUGAAGAACUG	GUUCUUCAACUGGCAGCUUUU
miR-22-3p inhibitor NC	CAGUACUUUUGUGUAGUACAA	/
miR-22-3p inhibitor	CAGUUCUUCAACUGGCAGCUU	/

**Table 2:** The information of qPCR primer.

Gene/miRNA Name	Forward Primer (5′-3′)	Reverse Primer (5′-3′)	Product length (bp)
*Actin*	GGCACCCAGCACGATGAAGA	GCATTTGCGGTGGACGAT	163
*GAPDH*	TCTCAAGGGCATTCTAGGCTAC	GCCGAATTCATTGTCGTACCAG	151
*IGFBP3*	CGCTACAAGGTTGACTACGAG	CAGTTGGGAATGTGGATGG	167
*IGFBP3*(full length)	CCCAAGCTTATATGCTGCGGGCACGCCC	CGGAATTCCTACTTGCTCTCCGTGCTGAGGCAG	882
*CDK2*	AGAAGTGGCTGCATCACAAG	TCTCAGAATCTCCAGGGAATAG	92
*PCNA*	CGAGGGCTTCGACACTTAC	GTCTTCATTGCCAGCACATT	97
*cyclin D1*	CCGAGGAGAACAAGCAGATC	GAGGGTGGGTTGGAAATG	91
*MyoG*	AATGAAGCCTTCGAGGCCC	CGCTCTATGTACTGGATGGCG	101
*MyoD*	GCTCCAGAACCGCAGTAAGTT	CGGCGACAGCAGCTCCATA	106
miR-22-3p	CGCGAAGCTGCCAGTTGAA	AGTGCAGGGTCCGAGGTATT	variable
*U6*	CTCGCTTCGGCAGCACA	AACGCTTCACGAATTTGCGT	95

Note. The underlined nucleotides indicate the restriction sites.

## Data Availability

Data can be acquired on reasonable request by contacting the corresponding author.
